# Supplementary material for: Two distinct binding modes provide the RNA-binding protein RbFox with extraordinary sequence specificity
Source: Nat Commun. 2023 Feb 9;14:701. doi: 10.1038/s41467-023-36394-3 (PMC9911399; doi:10.1038/s41467-023-36394-3)
Supplement: Supplementary file 1 — Supplemental Information [file 41467_2023_36394_MOESM1_ESM.pdf]

Supplementary Materials

Two distinct binding modes provide the RNA-binding protein RbFox with extraordinary sequence specificity

Xuan Ye, Wen Yang, Soon Yi, Yanan Zhao, Gabriele Varani, Eckhard Jankowsky, and Fan Yang

Twelve supplementary figures
One supplementary table

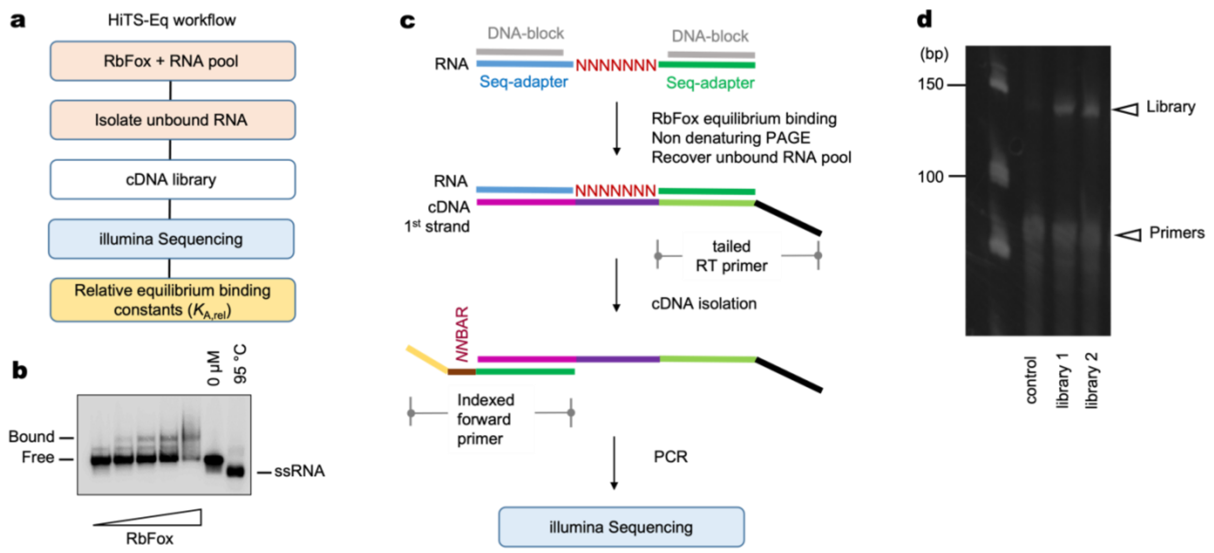

Supplementary Figure 1. HiTS-Eq approach.

a HiTS-Eq workflow. **b** Representative PAGE for RbFox binding to the randomized RNA pool. The unbound RNA species were recovered and converted into NGS library according to the HiTS-Eq protocol. Data were replicated independently three times with similar results. **c** Preparation of HiTS-Eq libraries for illumina sequencing (detailed information: Methods). **d** Representative PAGE of the HiTS-Eq libraries (expected length: 137 bp). Data were replicated independently three times with similar results. Source data are provided as a Source Data file.

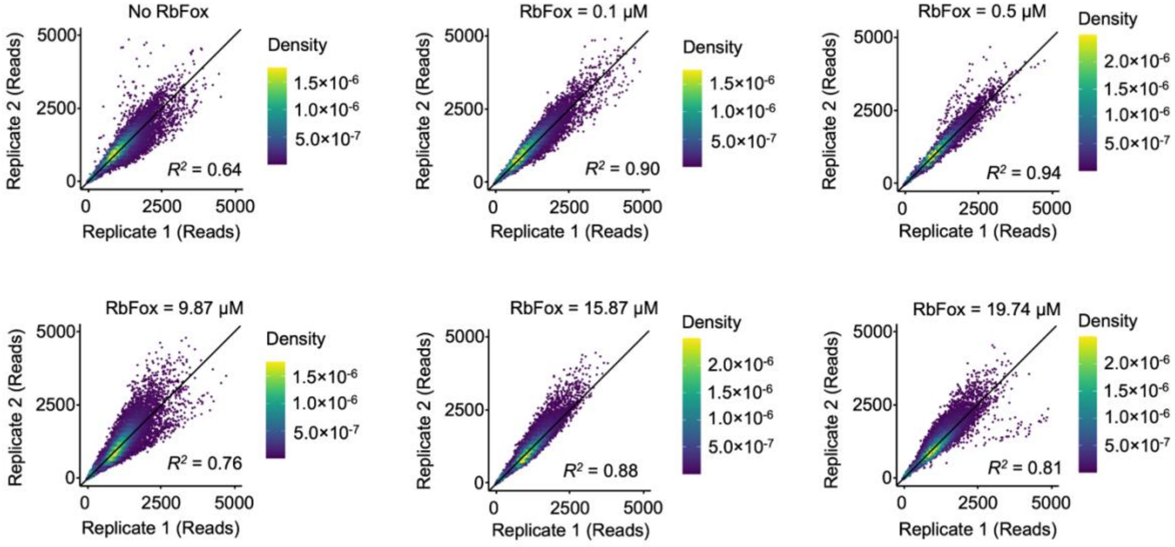

Supplementary Figure 2. Correlation between HiTS-Eq replicates.

Correlation between the two matched replicates for all 16,384 7-mer RNA sequence variants at different RbFox concentrations (line: diagonal, y = x; R^2, correlation coefficient). Source data are provided as a Source Data file.

a

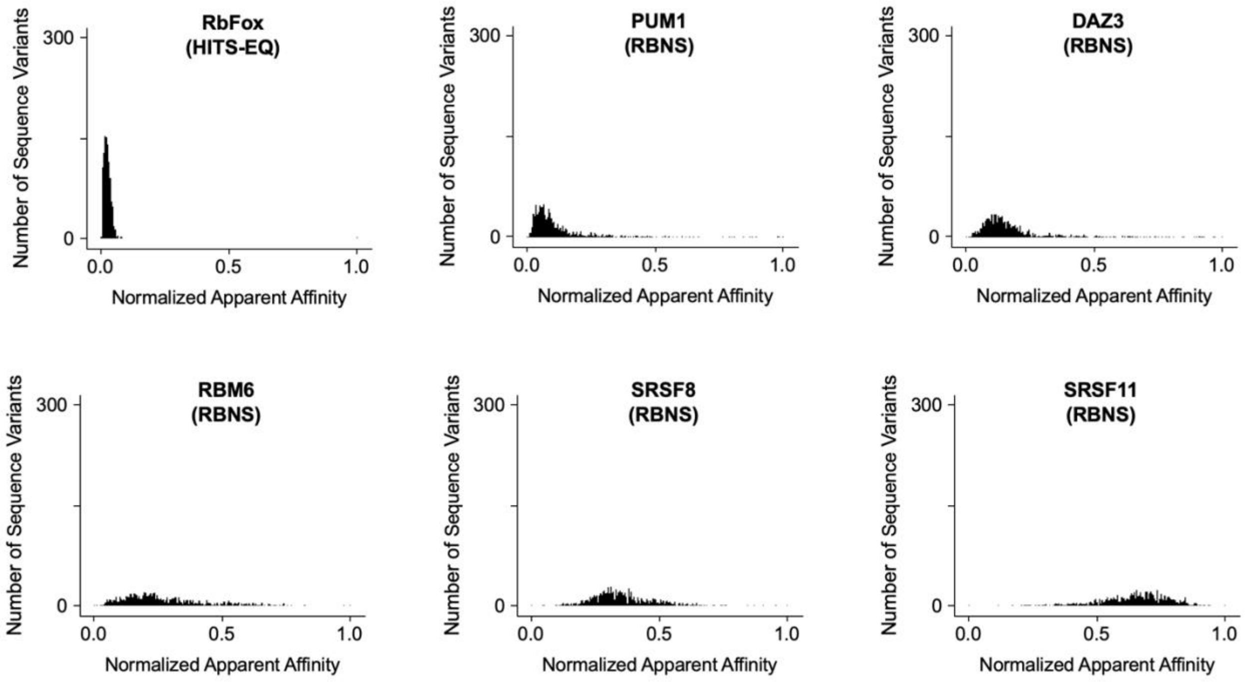

b

RBP	Affinity Ratio (max/median)
RbFox	31.5
PUM1	12.4
DAZ3	7.3
RBM6	4.3
SRSF8	3.0
SRSF11	1.5

Supplementary Figure 3. Affinity distributions and ratios for six RBPs.

a Global affinity distributions for six RBPs (RbFox, PUM1, DAZ3, RBM6, SRSF8, and SRSF11) from HiTS-Eq data and ENCODE RBNS datasets. **b** Affinity ratios between the highest affinity value to the median affinity value for the six RBPs. Source data are provided as a Source Data file.

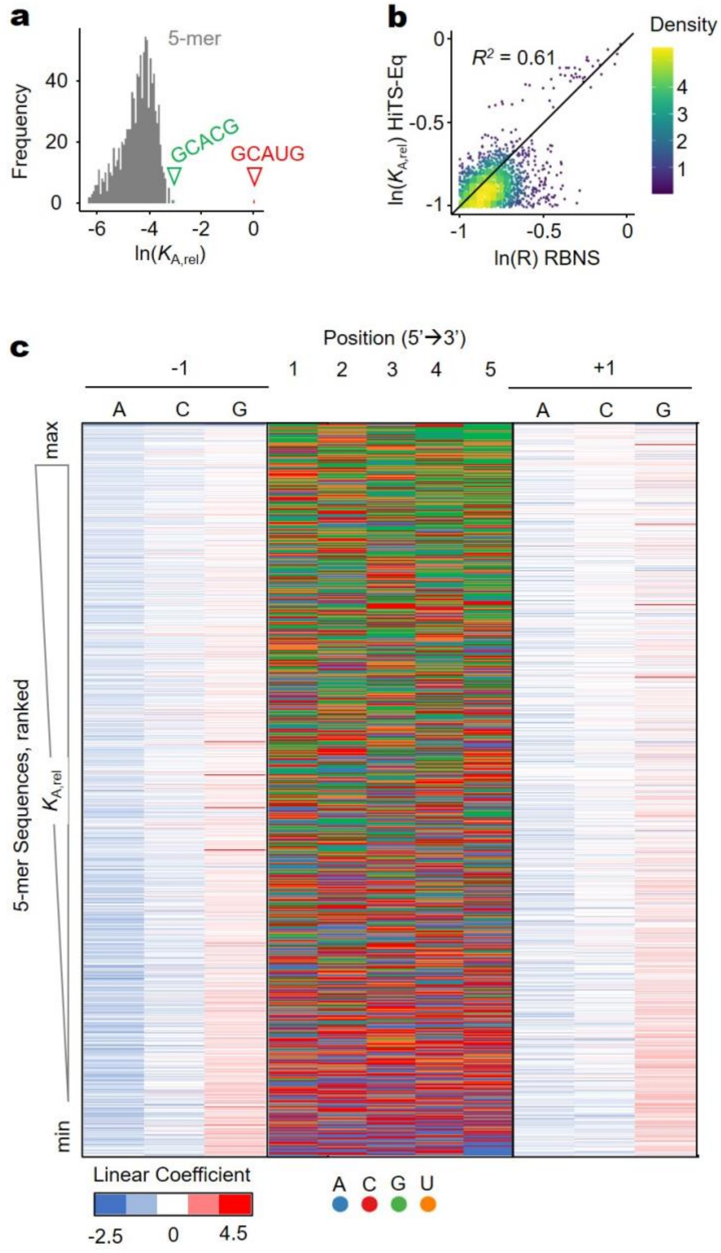

Supplementary Figure 4. RbFox binding to 5-mer RNA variants.

a Distribution of relative association constants ($K_{A,rel}$) of RbFox for all 1,024 5-mer RNA sequence variants (red triangle: reference GCAUG; green triangle: GCACG). **b** Correlation between relative association constants ($K_{A,rel}$) from the HiTS-Eq measurements and R value from RNA Bind-n-Seq (RBNS) measurements for all 1,024 5-mer RNA sequence variants. (line: diagonal, y = x; R^2, correlation coefficient). **c** Linear coefficient for -1 and +1 nucleotide position of all 1,024 5-mer RNA sequence variants calculated with the PWM binding model (negative values: destabilization). Source data are provided as a Source Data file.

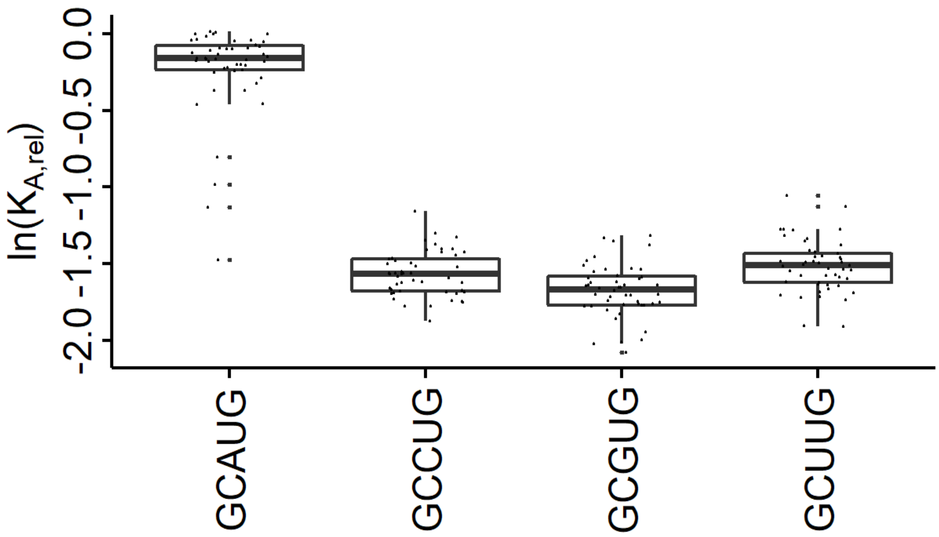

Supplementary Figure 5. Analysis of the binding affinities to RbFox for 5'-GCNUG sequences.

Relative affinities ($K_{A,rel}$) for selected 5-mer RNA variants, indicated on the below. 48 $K_{A,rel}$ values correspond to all 5-mer with 7 randomized nucleotides (vertical line: median; box: variability through lower quartile and upper quartile; whiskers: variability outside the lower and upper quartiles). Source data are provided as a Source Data file.

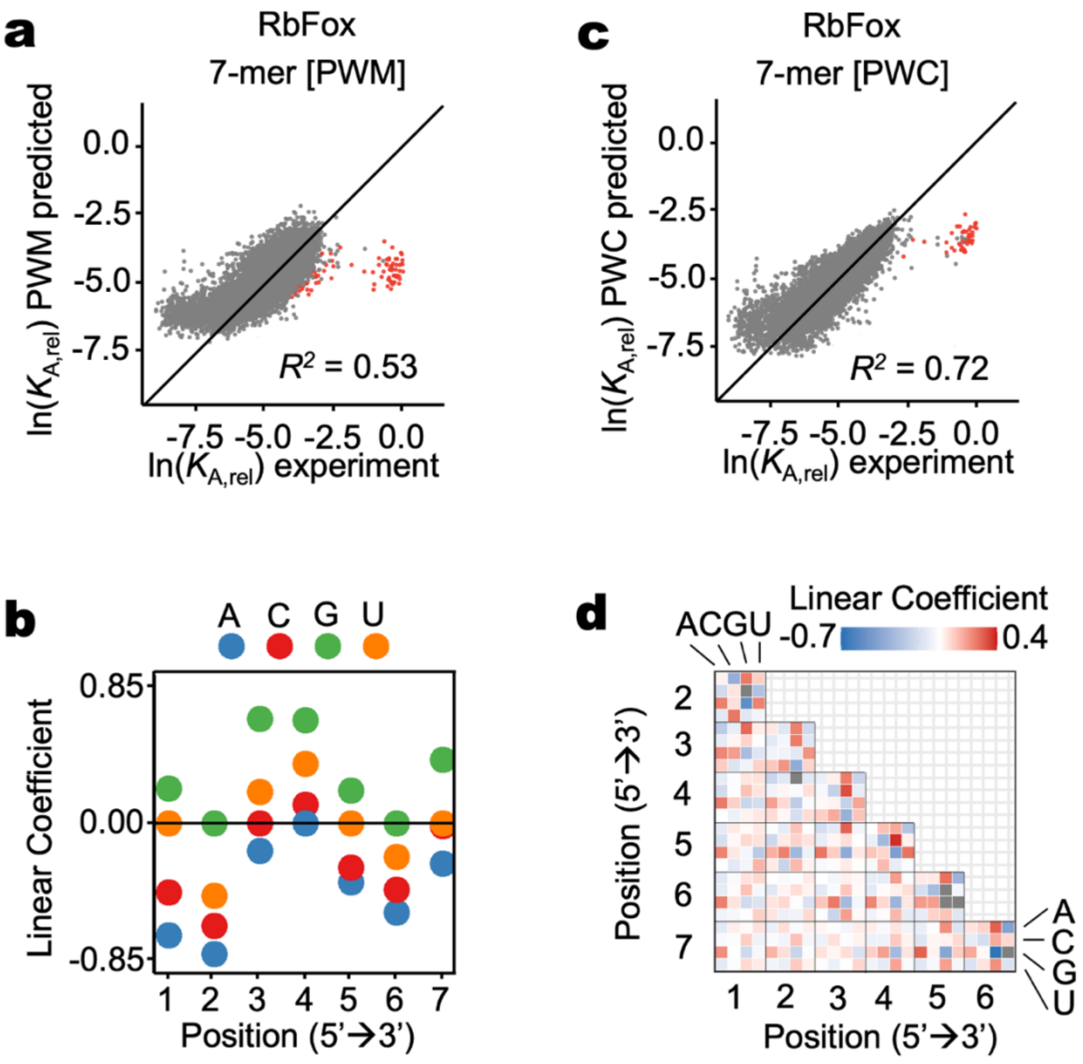

Supplementary Figure 6. Analysis of RbFox binding to 7-mer RNA variants with quantitative binding models.

a Correlation between experimental $K_{A,rel}$ values for RbFox for each 7-mer with values calculated with the PWM binding model; (red dots: consensus 5-mer; line: diagonal, y = x; R^2: correlation coefficient). **b** Linear coefficient for each nucleotide position calculated with the PWM binding model (negative values: destabilization). **c** Correlation between experimental $K_{A,rel}$ values for RbFox^{mut} for each 7-mer with values calculated with the PWC binding model (red dots: consensus 5-mer; line: diagonal, y = x; R^2: correlation coefficient). **d** Linear coefficients for each pairwise coupling between all nucleotides calculated with the PWC model; (black frames: couplings in consensus 7-mer, negative values: destabilization). Source data are provided as a Source Data file.

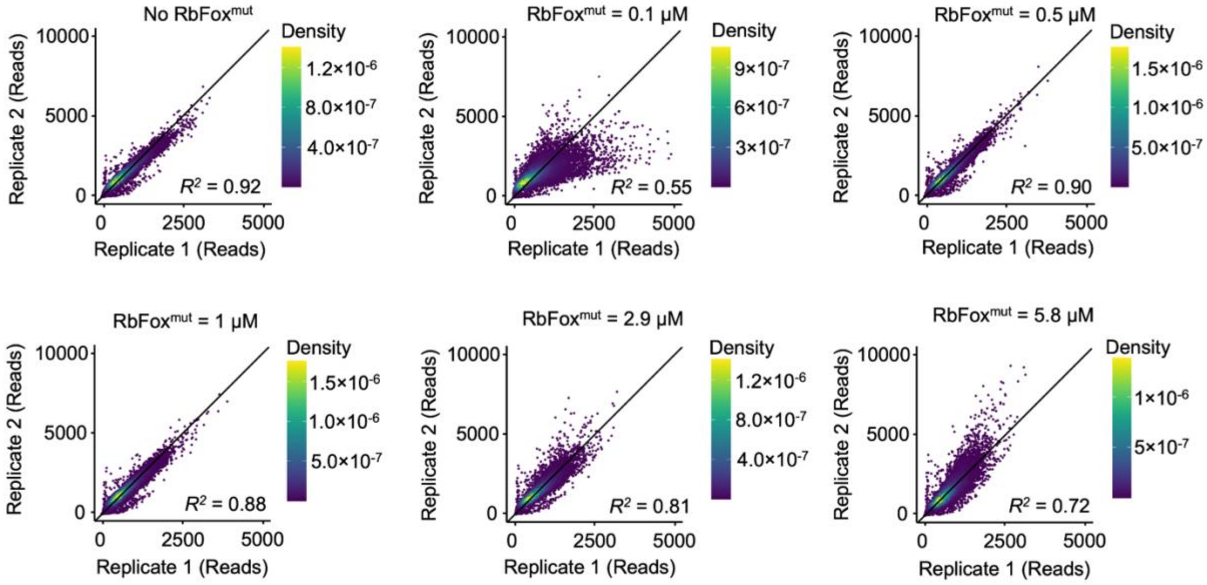

Supplementary Figure 7. Correlation between HiTS-Eq replicates for RbFox^{mut}.

Correlation between the two matched replicates for all 16,384 7-mer RNA sequence variants at different RbFox^{mut} concentrations; (line: diagonal, y = x; R^2, correlation coefficient). Source data are provided as a Source Data file.

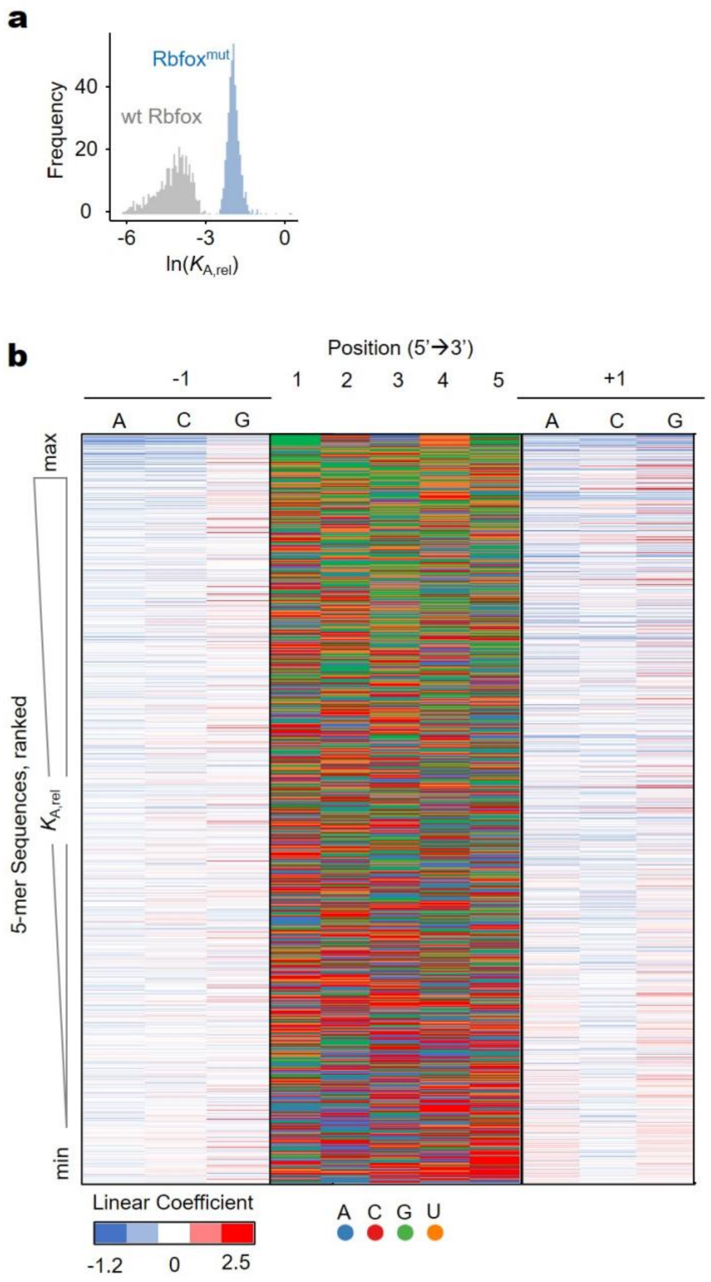

Supplementary Figure 8. RbFox^{mut} binding to 5-mer RNA variants.

a Affinity distribution ($K_{A,rel}$) of RbFox^{mut} for all 5-mer RNA sequence variants (blue); (bin size: 100). For reference, the affinity distribution of wild type RbFox (grey) is provided as well. **b** Linear coefficient for -1 and +1 nucleotide position of all 1,024 5-mer RNA sequence variants calculated with the PWM binding model (negative values: destabilization). Source data are provided as a Source Data file.

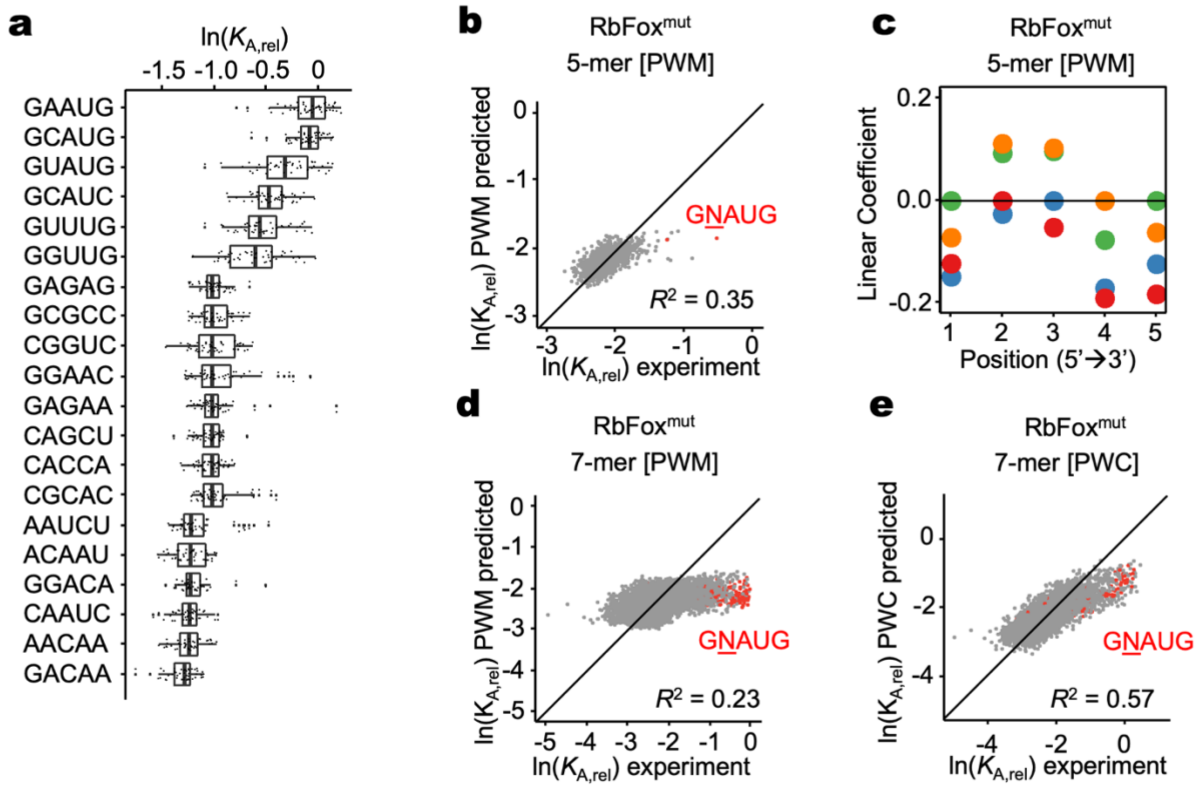

Supplementary Figure 9. Analysis of RbFox^{mut} RNA binding with quantitative binding models.

a Relative affinities ($K_{A,rel}$) for selected 5-mer RNA variants, as indicated on the left. 48 $K_{A,rel}$ values correspond to all 5-mer with 7 randomized nucleotides (vertical line: median; box: variability through lower quartile and upper quartile; whiskers: variability outside the lower and upper quartiles). **b** Correlation between experimental $K_{A,rel}$ values for each 5-mer (median value, panel **a**) and values calculated with the PWM binding model (red dots: consensus 5-mer; line: diagonal, y = x; R^2: correlation coefficient). **c** Linear coefficients for each nucleotide position calculated with the PWM binding model (negative values: destabilization). **d** Correlation between experimental $K_{A,rel}$ values for each 7-mer (median value, panel (A)) and values calculated with the PWM binding model (red dots: consensus 5-mer; line: diagonal, y = x; R^2: correlation coefficient). **e** Correlation between experimental $K_{A,rel}$ values for each 7-mer (median value, panel (A)) and values calculated with the PWC binding model (red dots: consensus 5-mer; line: diagonal, y = x; R^2: correlation coefficient). Source data are provided as a Source Data file.

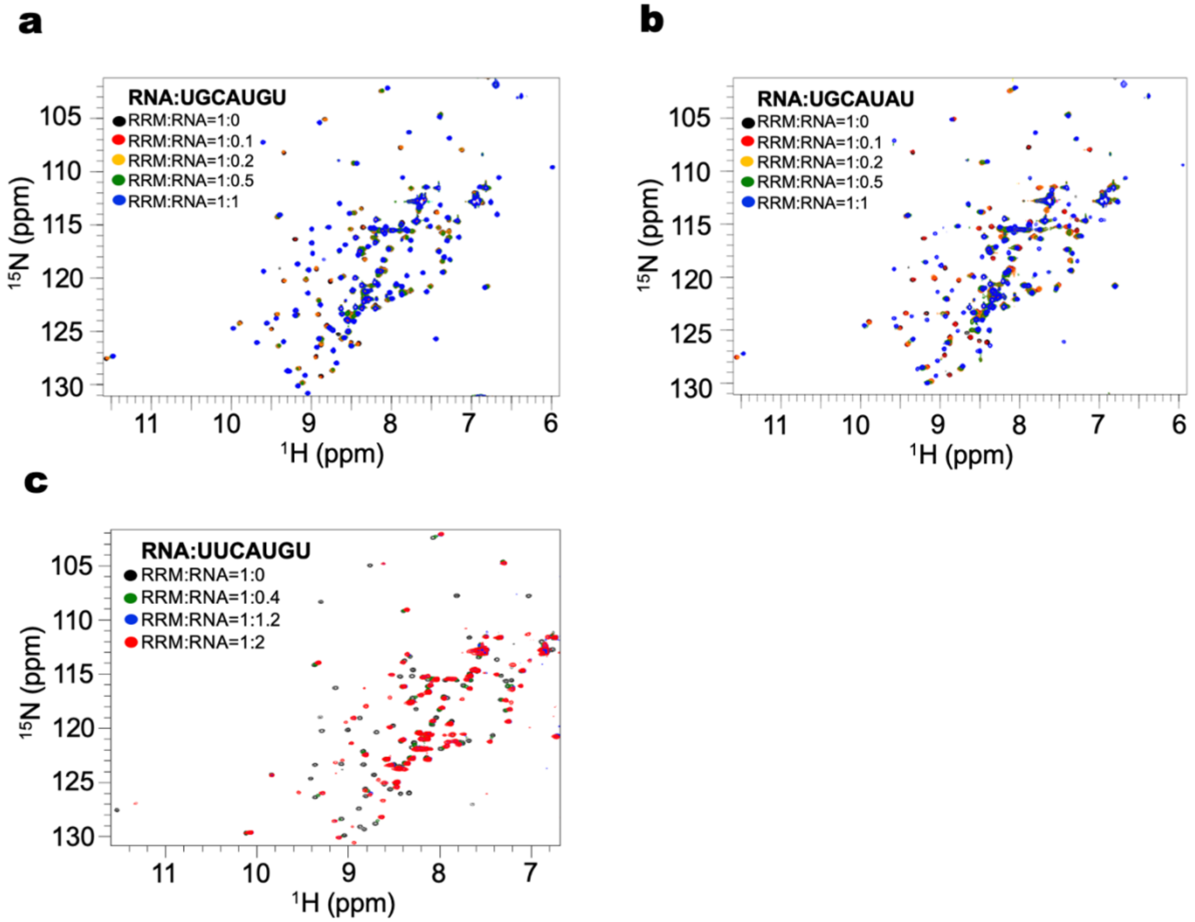

Supplementary Figure 10. ^{1}H-^{15}N HSQC titrations of RbFox RRM with three RNAs: 5'-UGCAUGU, 5'-UGCAUAU and 5'-UUCAUGU.

a Superposition of ^{1}H -^{15}N HSQC spectra obtained with ^{15}N-RbFox RRM and increasing amount of 5'-UGCAUGU RNA. The peaks corresponding to the free and RNA-bound RRMs (RRM:RNA ratios of 1:0, 1:0.1, 1:0.2, 1:0.5 and 1:1) are colored as black, red, orange, green and blue, respectively. **b** Superposition of ^{1}H -^{15}N HSQC spectra obtained with ^{15}N-RbFox RRM and increasing amount of 5'-UGCAUAU RNA. The color scheme is the same as in **a**. **c** Superposition of ^{1}H -^{15}N HSQC spectra obtained with ^{15}N-RbFox RRM and increasing amount of 5'-UUCAUGU RNA. The peaks corresponding to the free and RNA-bound RRMs (RRM:RNA ratios of 1:0, 1:0.4, 1:1.2, and 1:2) are colored as black, green, blue and red, respectively.

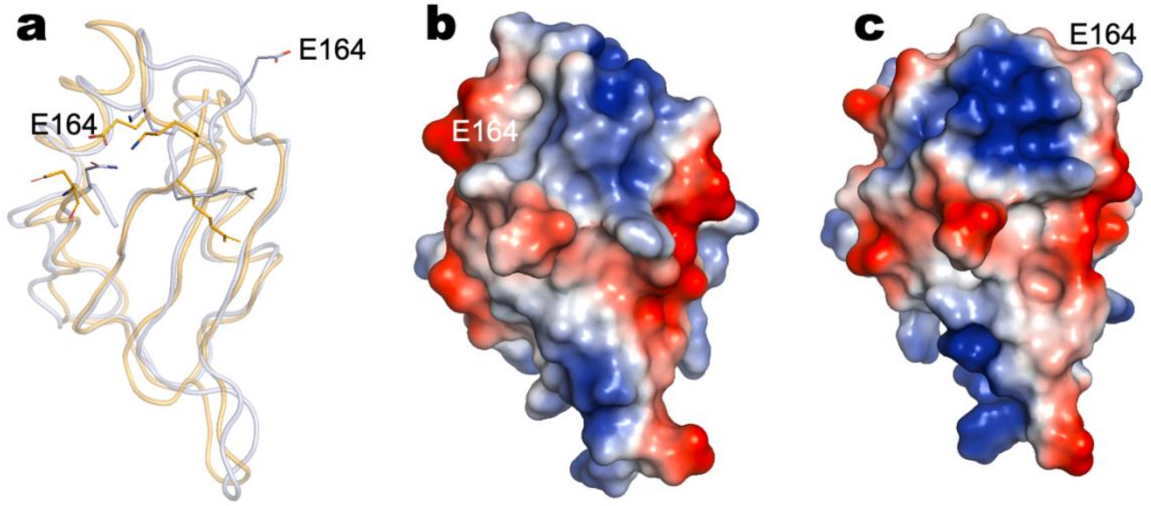

Supplementary Figure 11. Comparison of the surface electrostatics of the two RbFox-RNA complexes.

a Ribbon diagrams of the superposed RbFox structures bound to the different RNA (blue: complex with 5’-UGCAUGU; orange: complex with 5’-UGCAUAU). The side chain of the flipped out E164 residue which alters surface electrostatics through its relocation is explicitly labeled. **b,c** Surface electrostatics for the two RbFox-RNA complexes with 5’-UGCAUGU and 5’-UGCAUAU, respectively.

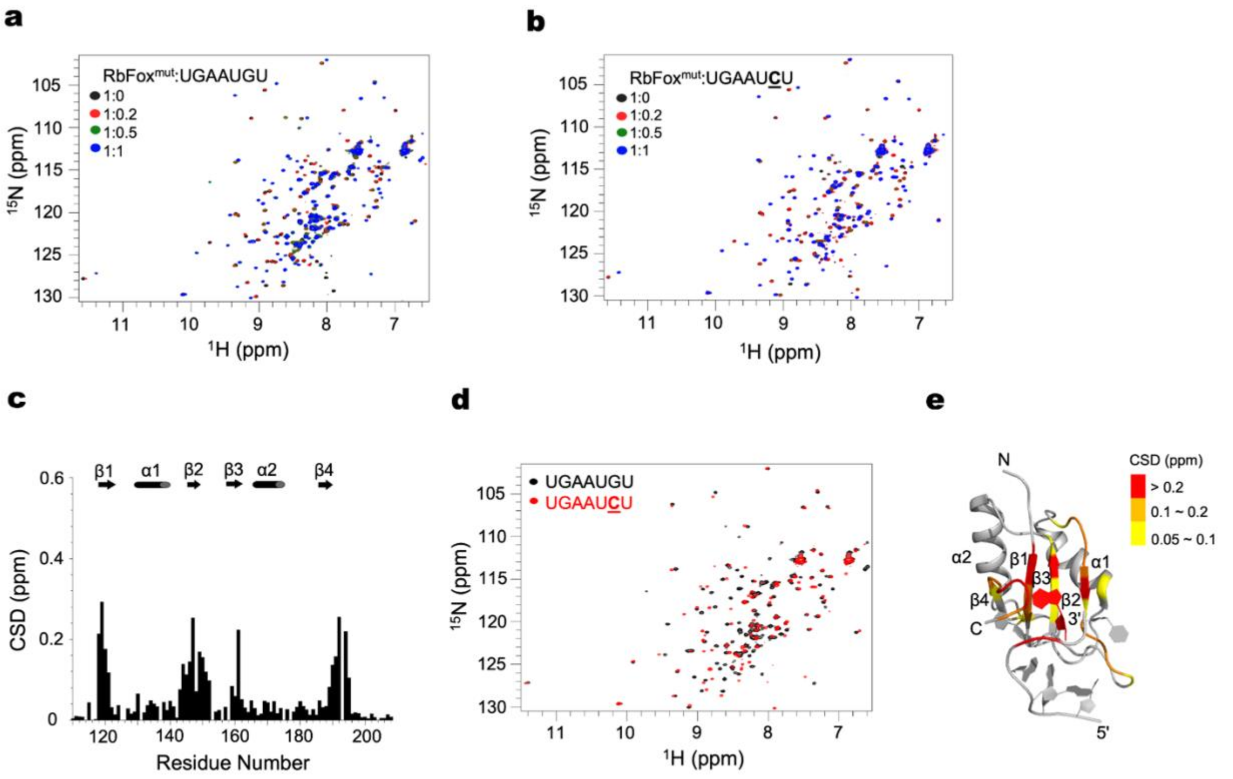

Supplementary Figure 12. Characterization of RbFox^{mut} bound to two RNAs: 5'-UGAAUGU and 5'-UGAAUCU.

a Superposition of ^{1}H -^{15}N HSQC spectra obtained with ^{15}N-RbFox^{mut} and increasing amounts of 5'-UGAAUGU RNA. The peaks corresponding to the free and RNA-bound RbFox^{mut}s (RRM:RNA ratios of 1:0, 1:0.2, 1:0.5 and 1:1) are colored as black, red, green and blue, respectively. **b** Superposition of ^{1}H -^{15}N HSQC spectra obtained with ^{15}N- RbFox^{mut} and increasing amounts of 5'-UGAAUCU RNA. The color scheme is the same as in **a**. **c** Chemical shift difference (CSD) between RbFox^{mut} bound to 5'-UGAAUGU and 5'-UGAAUCU. **d** Superposition of ^{1}H-^{15}N HSQC spectra of RbFox^{mut} complexed with the two RNAs (black: 5'-UGAAUGU; red: 5'-UGAAUCU). **e** Mapping of the CSDs in panel **c** onto the structure of the RbFox-RNA complex (pdb #2ERR) (red: CSD > 0.2 ppm; orange: 0.1 ppm < CSD < 0.2 ppm; yellow: 0.05 ppm < CSD < 0.1 ppm). Source data are provided as a Source Data file.

Supplementary Table 1. NMR structure statistics for RbFox RRM bound to 5'-UGCAUAU.

**Distance constraints**	
Total NOEs	2020
Protein-RNA intermolecular	130
RNA intramolecular	95
Protein intramolecular	1795
Protein intra-residue	421
Sequential (|i-j|=1)	480
Medium range (1<|i-j|<5)	287
Long range (|i-j|≥5)	607
Hydrogen-bond constraints	16
Torsion angle constraints	100
**Structure statistics (20 structures of lowest energy)**	
Violations	
NOE violations > 0.3 Å	0
Torsion angle violations > 5°	0
Ramachandran plot statistics	
Residues in most favored regions	82.0%
Residues in additional allowed regions	14.6%
Residues in generously allowed regions	2.2%
Residues in disallowed regions	1.1%
RMS deviations from the mean structure	
Protein backbone (Pro116-Arg194)	0.74
Protein heavy atoms (Pro116-Arg194)	1.20
RNA heavy atoms (G2-A6)	2.70
Complex heavy atoms (G2-A6 and Pro116-Arg194)	2.79
