# Supplementary material for: Two distinct binding modes provide the RNA-binding protein RbFox with extraordinary sequence specificity
Source: Nat Commun. 2023 Feb 9;14:701. doi: 10.1038/s41467-023-36394-3 (PMC9911399; doi:10.1038/s41467-023-36394-3)
Supplement: Supplementary file 3 — Reporting Summary [file 41467_2023_36394_MOESM3_ESM.pdf]

nature portfolio

Corresponding author(s): Gabriele Varani, Eckhard Jankowsky, Fan Yang

Last updated by author(s): Jan 17, 2023

Reporting Summary

Nature Portfolio wishes to improve the reproducibility of the work that we publish. This form provides structure for consistency and transparency in reporting. For further information on Nature Portfolio policies, see our Editorial Policies and the Editorial Policy Checklist.

Statistics

For all statistical analyses, confirm that the following items are present in the figure legend, table legend, main text, or Methods section.

n/a	Confirmed	
☐	☒	The exact sample size (*n*) for each experimental group/condition, given as a discrete number and unit of measurement
☐	☒	A statement on whether measurements were taken from distinct samples or whether the same sample was measured repeatedly
☒	☐	The statistical test(s) used AND whether they are one- or two-sided<br>*Only common tests should be described solely by name; describe more complex techniques in the Methods section.*
☒	☐	A description of all covariates tested
☒	☐	A description of any assumptions or corrections, such as tests of normality and adjustment for multiple comparisons
☐	☒	A full description of the statistical parameters including central tendency (e.g. means) or other basic estimates (e.g. regression coefficient) AND variation (e.g. standard deviation) or associated estimates of uncertainty (e.g. confidence intervals)
☒	☐	For null hypothesis testing, the test statistic (e.g. *F*, *t*, *r*) with confidence intervals, effect sizes, degrees of freedom and *P* value noted<br>*Give P values as exact values whenever suitable.*
☒	☐	For Bayesian analysis, information on the choice of priors and Markov chain Monte Carlo settings
☒	☐	For hierarchical and complex designs, identification of the appropriate level for tests and full reporting of outcomes
☒	☐	Estimates of effect sizes (e.g. Cohen's *d*, Pearson's *r*), indicating how they were calculated

Our web collection on statistics for biologists contains articles on many of the points above.

Software and code

Policy information about availability of computer code

Data collection	HiTS-Eq source code: https://github.com/hsuanchunlin/HiTS-EQ<br>HiTS-Eq source code backup: https://github.com/xxylO3/HiTS-EQ
Data analysis	Novocraft v3.0.8, Fastx-Toolkit v0.0.13, ImageQuant 5.2, KaleidaGraph v3.52, Amber 14.0, NMRPipe macll_64, CcpNmr Analysis 2.4.2, CYANA 3.98.13. PROCHECK v.3.5.4

For manuscripts utilizing custom algorithms or software that are central to the research but not yet described in published literature, software must be made available to editors and reviewers. We strongly encourage code deposition in a community repository (e.g. GitHub). See the Nature Portfolio guidelines for submitting code & software for further information.

Data

Policy information about availability of data

All manuscripts must include a data availability statement. This statement should provide the following information, where applicable:

- Accession codes, unique identifiers, or web links for publicly available datasets
- A description of any restrictions on data availability
- For clinical datasets or third party data, please ensure that the statement adheres to our policy

All data generated or analyzed in this study are included in the main text or the supplementary materials. Atomic coordinates and NMR data of RbFox complexed with 5'-UGCAUAU have been deposited in the Protein Data Bank under entry ID 7VRL [http://doi.org/10.2210/pdb7vrl/pdb] and Biological Magnetic Resonance

Bank under entry ID 36452 [https://dx.doi.org/10.13018/BMR36452]. One other published PDB code cited in this paper is 2ERR [https://doi.org/10.2210/pdb2err/pdb]. The source data are provided as a Source Date file with this paper.

Human research participants

Policy information about studies involving human research participants and Sex and Gender in Research.

Reporting on sex and gender	Not applicable.
Population characteristics	Not applicable.
Recruitment	Not applicable.
Ethics oversight	Not applicable.

Note that full information on the approval of the study protocol must also be provided in the manuscript.

Field-specific reporting

Please select the one below that is the best fit for your research. If you are not sure, read the appropriate sections before making your selection.

☒ Life sciences ☐ Behavioural & social sciences ☐ Ecological, evolutionary & environmental sciences

For a reference copy of the document with all sections, see nature.com/documents/nr-reporting-summary-flat.pdf

Life sciences study design

All studies must disclose on these points even when the disclosure is negative.

Sample size	We performed RbFox binding reactions for 16,384 randomized RNA variants using HiTS-Eq approach. For a 7-nt RNA, there are 16,384 sequence possibilities.
Data exclusions	We characterized comprehensive binding of RbFox to all RNA varaints and no data were excluded.
Replication	The biochemical characterizations described in Fig. lb, If, 3c, 3d were performed in biological triplicates with similar results. The HiTS-Eq experiments studied in Fig. 1g, 3e, Supplementary Fig. 4, 8 were conducted in two biological replicates with similar results. NMR titrations in Fig. 5 and Supplementary Fig. 10, 12 were replicated three times with similar results.
Randomization	Not applicable, because there is no specific grouping in data collection or analysis.
Blinding	Not applicable, because there is no specific grouping in data collection or analysis.

Reporting for specific materials, systems and methods

We require information from authors about some types of materials, experimental systems and methods used in many studies. Here, indicate whether each material, system or method listed is relevant to your study. If you are not sure if a list item applies to your research, read the appropriate section before selecting a response.

Materials & experimental systems

n/a	Involved in the study	
☒	☐	Antibodies
☒	☐	Eukaryotic cell lines
☒	☐	Palaeontology and archaeology
☒	☐	Animals and other organisms
☒	☐	Clinical data
☒	☐	Dual use research of concern

Methods

n/a	Involved in the study	
☒	☐	ChIP-seq
☒	☐	Flow cytometry
☒	☐	MRI-based neuroimaging
